# Clinical Effectiveness, Access to, and Satisfaction with Care Using a Telehomecare Substitution Intervention: A Randomized Controlled Trial

**DOI:** 10.1155/2011/540138

**Published:** 2011-12-01

**Authors:** Kathryn H. Bowles, Alexandra L. Hanlon, Henry A. Glick, Mary D. Naylor, Melissa O'Connor, Barbara Riegel, Nai-Wei Shih, Mark G. Weiner

**Affiliations:** ^1^University of Pennsylvania School of Nursing, 418 Curie Boulevard, Philadelphia, PA 19104, USA; ^2^University of Pennsylvania School of Medicine, 423 Guardian Drive, Philadelphia, PA 19104, USA

## Abstract

*Background*. Hospitalization accounts for 70% of heart failure (HF) costs; readmission rates at 30 days are 24% and rise to 50% by 90 days. Agencies anticipate that telehomecare will provide the close monitoring necessary to prevent HF readmissions. *Methods and Results*. Randomized controlled trial to compare a telehomecare intervention for patients 55 and older following hospital discharge for HF to usual skilled home care. Primary endpoints were 30- and 60-day all-cause and HF readmission, hospital days, and time to readmission or death. Secondary outcomes were access to care, emergency department (ED) use, and satisfaction with care. All-cause readmissions at 30 days (16% versus 19%) and over six months (46% versus 52%) were lower in the telehomecare group but were not statistically significant. Access to care and satisfaction were significantly higher for the telehomecare patients, including the number of in-person visits and days in home care. *Conclusions*. Patient acceptance of the technology and current home care policies and processes of care were barriers to gaining clinical effectiveness and efficiency.

## 1. Introduction

Heart failure (HF) is the most common chronic illness in home care, affecting almost six million Americans today. HF costs $39.2 billion annually in the US [1], with hospitalizations accounting for 70% of HF costs [2]. Readmission rates at 30 days are 24% nationwide [3] and rise to 50% by 90 days [4–6], though half of these readmissions may be preventable [6]. With a hospital fatality rate of 51% [1], and with each hospitalization costing $7,174–$10,000 [7], it is imperative to prevent or reduce readmissions.

Symptoms of heart failure exacerbation, such as weight gain, edema, and increased dyspnea, frequently present 8 to 12 days before admission [8]. Using daily remote monitoring these symptoms can be recognized and treated before readmission becomes necessary. Increasing incidence and prevalence of HF [9], “quicker and sicker” hospital discharges [10], and the current nursing shortage [11] make it challenging to efficiently provide the necessary close monitoring and teaching that HF patients require.

Telehomecare is defined as a communication and clinical information system that enables the interaction of voice, video, and health-related data using ordinary telephone lines from the patients' homes in conjunction with home visits [12]. The intent is for patients to use the equipment to self-monitor, then actively partner in collaboration with health care providers to manage symptoms. The evidence to date on the cost and clinical effectiveness of telehomecare monitoring is mixed and difficult to interpret as a body of evidence [2, 8, 10, 13–25]. Most studies have small sample sizes and lack standardization of the intervention making it difficult to synthesize results. 

All previous studies provided telehomecare in addition to usual care, leading our team to question if using it as a substitute for some routine home visits might increase its efficiency. This study was the first attempt to test the effects of telehomecare when used in collaboration with the patient in place of some in-person skilled nursing visits. Many authors claim that telehomecare is capable of maintaining quality of care while creating potential for cost savings [26] through higher patient : nurse ratios [27, 28] and decreased travel costs [29, 30]. Others say it may be possible to conduct about 45% of home care visits through telehomecare, yielding savings of up to $700 per patient [31, 32]. Based on these estimates, 15–25 patients a day can access care via video telehomecare technology versus, on average, a driving visiting nurse can only see 5-6 patients per/day. Agencies that utilize telehomecare actually increase their patient's access to care by performing standard in-home visits, remotely monitoring, and allowing patients to transmit data as often as they choose. The challenge lies in achieving the balance between meeting patient care goals and efficiency. 

The purpose of this study was to compare the effects of a telehomecare intervention that substitutes for 45% of in-person skilled nursing visits on time to readmission, readmission rates, hospital days, emergency department use, access to care, and satisfaction for older adults following hospital discharge for HF to standard skilled home care services.

## 2. Methods

### 2.1. Design

This study was a randomized, controlled clinical trial conducted as a field study with 217 HF patients in a not-for-profit home care agency in Philadelphia. The intervention group (*N* = 101) received home care consisting of a combination of in-person and telehomecare daily monitoring and intermittent video visits. The type and number of visits were guided by a standardized study protocol that defined minimal expectations of at least four video visits and daily use of the devices over the home care episode and at least 5 in-person home visits for either group. The goal was for the home care nurse to collaborate with the patient to use the technology in place of 45% of the skilled nursing in-person visits. The intervention occurred over an episode of home care, from the initial start of care until officially discharged from the home health agency. Outcomes were assessed at 30, 60, 120, and 180 days. All of the nurses involved in caring for both standard care/control and intervention subjects were guided by the agency's evidence-based clinical pathway for HF management. Both groups aimed to monitor and teach patients how to self monitor and manage their needs following discharge from home care, but the standard care nurses and patients (*n* = 116) did so without the technology. 

### 2.2. Sample

The sample was drawn from all patients 55 and older discharged from area hospitals within two weeks of enrollment for HF as a primary or secondary diagnosis and referred to home care with the study agency. Patients in another HF clinical trial, on dialysis, on the heart transplant list, with cancer as a primary diagnosis, or in disease management were excluded. Eligible patients spoke English; were mentally competent as determined by the Mini Cog test [33, 34]; weighed less than 450 pounds (scale maximum); had a land line telephone; were able to see, hear, place a cuff on their arm, and stand on a scale to weigh themselves; and were eligible for, referred to, and accepted home care services. The protocol was approved by the university institutional review board and written informed consent was obtained before randomization. 

Power calculations were based on home care agency reported 60-day readmission rates of 37% at the time of study planning. Unpublished pilot work and experience in other studies indicated that the readmission rate may decrease to 19% using telehealth [35, 36]. Thus, if the readmission rate in the telehomecare group is half that of the usual care, the anticipated difference was 19% with alpha = 0.05 yielding power of  0.80. This would require a sample size of 90 per group (or 180 total subjects). To account for the anticipated effect of a 20% attrition rate, we targeted 108 subjects per group for a total of 216 subjects. The principle investigator and research assistants remained blinded to study group. It was not possible to blind the patients or the home care nurses since they had to interact with the telehealth equipment. Enrolled patients were randomized by the project manager using an allocation spreadsheet prepared by the statistician using a randomly permuted blocks algorithm to insure equal distribution between the two groups. The sample was stratified on length of time since HF diagnosis: less than two months versus more than or equal two months based on research that demonstrated significant differences in HF self-care between patients diagnosed less than two months and those diagnosed more than or equal two months [37]. Enrollment began in March 2006 and ended in November 2009. 

### 2.3. Procedures

Patients assigned to the telehomecare group received telehealth equipment in their homes and nurses followed the intervention group protocol described below. The home care nurse manager and visiting nurse at the home care agency were notified of patient group assignment; so the nurse knew to expect the arrival of telehomecare equipment and to follow the telehomecare protocol versus usual care. Usual home care consists of at least five intermittent in-person skilled visits by a registered nurse over a 60-day episode to assess, teach, and case manage the patient's care. 

Two education sessions were held with all the agency field nurses and their managers at the start of the study with six refresher sessions conducted throughout the course of the study in addition to personal sessions by the project manager as needed. All agency nurses (*N* = 52) attended the education sessions to learn about heart failure management, how to apply the clinical pathway, install and operate the telehealth equipment, teach patients how to use the equipment, and details about the study protocol to guide substitution of telehomecare visits for in-person visits. Randomization of the nurses was not possible because they are assigned to patients by geographic area and cover for each other on days off and vacations. Logistically it would have impaired the agency operations. Therefore, the patients were randomized and the same nurses cared for patients in either group and used the clinical pathway to guide the care whether the patient was receiving telehealth monitoring or not. This was instituted to ensure a consistent baseline of standard care; so the telehealth technology and substituted visit pattern become the only difference between the groups. The pathway guided the schedule of assessments and teaching topics for each week of the home care episode. Over the course of the study 28 nurses among the 52 trained cared for study patients as they were admitted in their geographic locations. None of the nurses had prior experience with telehomecare. 

Once the patient was randomized to group, the project manager ordered the telehealth equipment based on patient need. For example, if they had diabetes or chronic obstructive pulmonary disease in addition to HF, they received a glucometer and pulse oximeter, respectively. All patients received a video phone, blood pressure cuff, and a weight scale. The equipment was shipped to the patients' home within the first week of home care and installed either by the patient and family or by the home care nurse on the first visit. Installation success was tested remotely by the project manager. The devices were wireless for easy placement throughout the home and transmitted data via a hub automatically every day that connected to the Internet via a telephone line. Nurses taught the patients and their caregivers how to operate the equipment and reviewed the study goals. Patients were taught to use the devices daily by 11 am and a telehealth nurse at the agency monitored the data daily for out of range readings. The devices were supplied by Carematix, Inc. Chicago, Illinois. According to protocol, the telehomecare nurse was to make at least four video visits with the patient in addition to their daily monitoring. Video visits were considered important for teaching and to replace personal contact as home visits were decreased. The home care nurses conducted the in-person home visits and four telehomecare nurses monitored the data and conducted the video visits. The telehomecare nurses notified the home visiting nurses and/or patient via phone or voicemail if readings were out of normal range to obtain changes in the treatment plan or confirm the accuracy of the transmission with another reading (i.e., blood pressure) or assessment of other symptoms (shortness of breath). The telehomecare nurses and visiting nurses collaborated on the plan of care and determined when to notify a physician of symptoms or changes in the measures. It was not feasible for the telehomecare nurses and the home visiting nurses to be the same person because the home care nurses did not travel back to the office which was required to conduct the video visits. Once out in the field visiting it would not be cost effective to have them drive in to do a video visit. It would also have been difficult to schedule.

The project manager gave a suggested visit pattern to each nurse to guide the substitution pattern (Table 1). A rigorous process was followed to encourage that the visit protocol was followed starting with education, six refresher classes, monitoring by the project manager, and one-on-one communication with the nurses. The visit protocol was provided to every nurse caring for a telehealth patient in a folder upon admission. The project manager monitored the visit pattern and was in contact with the nurses throughout the home health episode encouraging that the visit protocol be followed. For the occasions when it was detected that the visit pattern was not being followed, the project manager contacted the nurse directly to discuss why and reviewed the study protocol as well as notified the nurse's manager. The refresher inservices and the project manager's individualized coaching of field nurses were in addition to periodic contact between the principal investigator and agency executive administration in an attempt to obtain the support needed for protocol adherence. 

### 2.4. Data Collection and Outcome Measures

Consenting patients were interviewed at baseline (enrollment day 0) in person or by telephone by trained and blinded bachelor's nursing student research assistants and by telephone at 60, 120, and 180 days. Information about readmissions, ED use, and length of stay was also collected from the hospital administrative database and medical records departments. 

#### 2.4.1. Health Care Utilization

The effects of the telehomecare intervention were measured on the primary outcome health care resource utilization at 30 and 60 days which included length of time to first rehospitalization or death, numbers of all-cause and heart failure-related rehospitalizations, hospital days, and emergency department visits. The health care utilization information was collected from the home care agency records, the health system administrative database, and patient interview. All readmissions occurring outside of the health system were confirmed through hospital medical record departments. Data were collected on the date, reason, location, length of stay, and cost of all readmissions.

#### 2.4.2. Access to Care

Access to care was defined as the amount of patient-provider contact via in-person home visits and telehomecare visits. This was collected from the home care agency records.

#### 2.4.3. Patient Satisfaction

Satisfaction with home care was measured by telephone survey upon discharge from home care by the blinded research assistants. The survey was designed specifically for home health care patients and was tested in a sample of 696 patients from thirteen home health agencies in Pennsylvania and Ohio. Construct validity was obtained in a pilot test using home health nurses. The reliability of the scale was  0.94. The tool measures aspects of scheduling, nursing interventions and relationships, discharge plans, and general measures of satisfaction [38]. It did not specifically address telehealth but rather was an assessment of satisfaction with home care services. Response categories ranged from 1 to 5, with higher scores indicating higher levels of satisfaction. The survey takes approximately 10 minutes to administer.

### 2.5. Data Analysis

All analyses were conducted using an intent-to-treat approach, such that all randomized patients are analyzed in the treatment groups to which they were randomly assigned. Subjects having at least one follow-up data point qualified for inclusion.

Descriptive statistics were used to characterize demographic and clinical characteristics, with means/standard deviations representing continuous measures and frequencies/percents representing categorical variables. Comparisons by intervention group were examined using chi-square statistics and two-sample *t*-tests for categorical and continuous variables, respectively. Covariates demonstrating imbalance at the 0.10 level of significance were included in subsequent multivariate modeling. 

#### 2.5.1. Time to Rehospitalization or Death

Time to first rehospitalization or death was measured from the date of the index hospitalization discharge to the date of first rehospitalization or death. Patients alive and remaining free from hospitalization were censored at their last follow-up interview date. A multivariate analysis of time to first rehospitalization or death was accomplished using Cox proportional hazards regression modeling, with outcome regressed on intervention group and adjusted for covariates emerging significant on bivariate analysis [39]. Hazard ratios are provided for intervention and covariates, along with their 95% confidence intervals. The proportional hazards assumption was examined and satisfied [40]. Kaplan Meier estimates were generated and used to visually demonstrate time to first rehospitalization or death by intervention group; the log-rank test was used for univariate intervention group comparisons [41, 42]. 

#### 2.5.2. Number of All-Cause and Heart Failure-Related Readmissions, Hospital Days, and ED Visits

Poisson GEE [43] models (log link) were used to model the total number of rehospitalizations and hospital days through 180 days, with an offset term of the natural logarithm of days included to control for varying days at risk for rehospitalization over the time period based on hospitalization or death. A working exchangeable correlation matrix was used to model repeated observations on the same person. To evaluate group differences in outcome over time, and thus explore temporal intervention effects, independent variables initially included intervention group, time (30, 60, 120, 180 days), and a time-by-group interaction term, as well as important covariates emerging significant in preliminary analyses. Subsequent models were based on main effects only (group, time, covariates), given that changes over time by group were clinically and statistically insignificant. Exponentiated parameter estimates can be interpreted as rate ratios (RRs), estimating the relative change in incidence rate associated with differences in level for categorical predictors, or with a one unit change in the independent variable for continuous variables.

#### 2.5.3. Access to Care

Total number of home visits and length of stay in home care were calculated from index home care admission to discharge. Patients kept on service for another episode of home care were counted as recertified and their visits and days were included in the total visit count and length of stay. Patients that accepted versus refused the telehealth equipment were compared on severity of illness; time with HF; number of medications, previous hospitalizations in six months, and comorbid conditions; and socio-demographics (race, gender, income). The number of video visits conducted was summarized and the percent of telehealth use was calculated as the number of days the patient used the equipment divided by the number of days the equipment was in the home ×100. Chi square or *t*-tests were used to compare the groups on characteristics of those who refused versus accepted telehealth. The independent sample Mann Whitney *U*-test was used to compare the number of home visits, mean number of visits over the study period, and the length of stay in home care. The two-sided Fishers Exact test was used to assess the association between group and recertification.

#### 2.5.4. Patient Satisfaction

Descriptive statistics and chi square were used to compare satisfaction scores between the usual care group and the telehomecare group. 

## 3. Results

**Figure 1:**
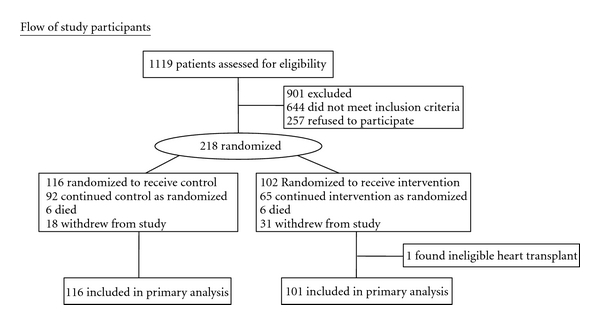
Consort diagram.

Figure 1 shows the consort diagram where 1,119 patients were screened, 644 were found ineligible (58%), and 257 refused to participate (23%). The most frequent reasons for ineligibility were no referral to home care (18%), unable to reach for enrollment prior to two weeks after discharge (16%), cognitive impairment (11%), or unable to hear well enough on the phone (6%). Two hundred and eighteen patients were enrolled in the study and randomly assigned by the project manager. The stratification based on length of time in months with heart failure was successful with the control group mean at 61.4 months, SD = 71.6 and telehomecare patients at 60.1 months, SD = 67.6, *P* = 0.81. One patient randomized to the telehomecare group was removed before analysis because he/she was found to be ineligible (on the heart transplant list and transplanted), making the final sample 217, usual care 116, and telehomecare 101.

Of the 101 patients enrolled in the telehomecare group, 36 (36%) did not receive any dose of telehomecare but were still included in the intent to treat analysis. Of these, 24 refused the equipment upon arrival, eight were discharged from home care before delivery, three changed their minds about being in a study, and one died before delivery. Reasons for non acceptance of the equipment included patients being “too sick to bother”, or some expressed concern over nurses altering their phone systems to connect the equipment, and two refused because the nurses discouraged them from participating since the nurse had to set up the equipment. Younger patients were significantly more likely to accept the technology than older patients (mean age 69, SD 10.6 versus 74.5, SD 8.5, *P*  0.015). 

Withdrawals (*N* = 31) in the telehomecare group include the 24 who refused the equipment upon arrival and seven who withdrew later (31%) compared to 18 (16%) withdrawals in the control group. Only one patient was lost to follow-up in the telehomecare group because he/she was discharged early from home care, while in the control group 15 patients (13%) were unable to be reached after enrollment. All subjects were followed for six months. Five patients died in the control group (4.3%) and four in the telehomecare group (3.9%), (*P* = 0.822). Although patients withdrew or were lost to follow-up for phone interviews, data on the primary health care utilization outcomes were available for all study subjects from agency databases; so missing data are not an issue for the primary outcomes. 

### 3.1. Sociodemographic and Clinical Comparisons

Table 2 shows the socio-demographic and clinical characteristics of all enrolled patients at baseline. Telehomecare patients were taking significantly more medications, mean 11.3, SD 4.6, compared to mean of 10.0, SD 3.4 for control patients, *P* = 0.020. Telehomecare patients were younger, mean age 71.3 (SD = 10.2) versus 73.5 (SD = 9.6) for control patients, *P* = 0.092. All analyses were adjusted for these differences. 

Overall, study patients had high-risk characteristics such as 69% rated their health as fair or poor, 32% had less than a high school education, 65% were African American, 39% had an annual income <$20,000/year, 69% were hospitalized at least twice in the 12 months prior to enrollment, and 34% lived alone and had an average of 6.4 comorbid conditions. 

### 3.2. Time to First Readmission or Death

**Figure 2:**
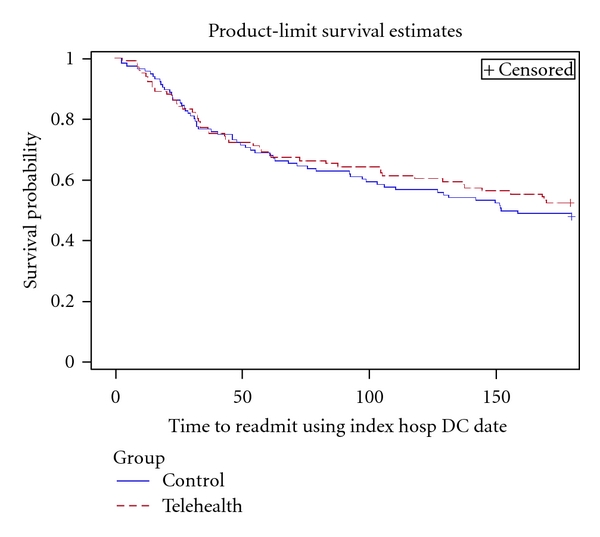
Kaplan-Meier survival curve of time to first all-cause readmission or death for telehealth group versus control usual care group (LR *P*-value = 0.585).

In Figure 2 the Kaplan-Meier Survival Curve and log rank test shows that there is no significant difference in time to readmission or death between intervention and control group (log-rank *P* = 0.585). A Cox proportional hazards model was used to compare intervention and control group for the time to first readmission adjusting for age and number of medications.  Table 3 shows that there is no significant difference between the two groups (*P* value = 0.319).

### 3.3. Number of All-Cause and Heart Failure-Related Readmissions

By 30 days, 19% of control group patients and 16% of telehomecare patients had at least one all cause readmission; however this 3% difference was not statistically significant (*P* = 0.546). By six months 52% (*n* = 60) versus 46% (*n* = 46) of control versus intervention patients were readmitted at least once (Table 4). The difference in overall percent of patients experiencing readmissions over six months is 6% and remained statistically insignificant, with *P* = 0.363, but clinically relevant with telehomecare patients having fewer readmissions.

Similarly, there were no significant differences in the number of heart failure-related readmissions (Table 4). For example, by 30 days 9% of usual care patients were readmitted for heart failure and 8% of telehomecare patients. Overall, including 0–30 days, 31–60 days, 61–120 days, and 121–180 days there were no significant differences by group after controlling for age and number of medications (*P* = 0.230, 95% CI 0.536 to 1.161) for all-cause and heart failure-related readmissions (*P* = 0.977, 95%  CI = 0.555–1.769) (Table 5). 

### 3.4. Number of All-Cause and Heart Failure-Related Hospital Days

Mean number of hospital days for all-cause readmissions occuring 0–30 days after index discharge was 1.41 ± 4.05 for usual care and 0.91 ± 2.49 for telehomecare patients (Table 6). Mean number of hospital days for heart failure-related readmissions for same time period was 0.38 ± 1.38 for control versus 0.48 ± 1.75 for telehomecarepatients (Table 6). If readmitted, the hospital days between the telehomecare and usual care groups showed no significant difference for all-cause (*P* = 0.260, CI = 0.468–1.227) and heart failure-related stays (*P* = 0.523, CI = .634–2.44) by six months (Table 7). 

### 3.5. Time to Emergency Department Use

**Figure 3:**
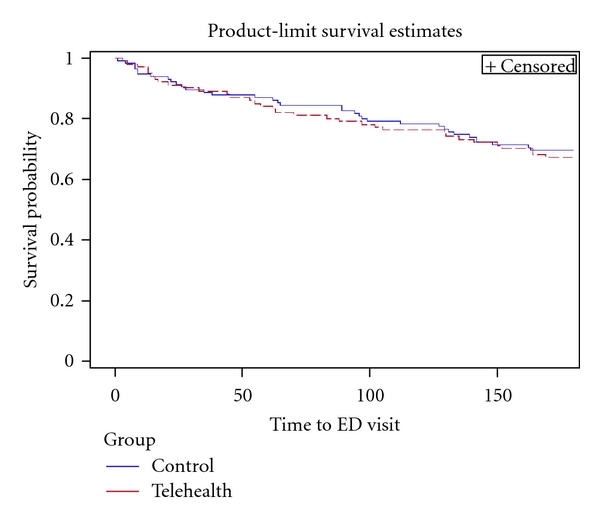
Kaplan-Meier survival curve of time to ED use for telehealth group versus control usual care group (LR *P*-value = 0.699).

In Figure 3 the Kaplan-Meier Survival Curve and log rank test shows that there is no significant difference in time to ED use between intervention and control group (log-rank *P* = 0.699). A Cox proportional hazards model was used to compare intervention and control group for the time to first readmission adjusting for age and number of medications and found no significant difference, *P* = 0.231 (Table 8). 

### 3.6. Emergency Department Use

By 30 days, 10% of control group patients and 10% of telehomecare patients had at least one ED visit (*P* = 0.939). By six months 30% (*n* = 35) versus 33% (*n* = 33) of control versus telehomecare patients used the ED (*P* = 0.832) (Table 9). Overall, including 0–30 days, 31–60 days, 61–120 days, and 121–180 days there were no significant differences by group for emergency department visits after controlling for age and number of medications (*P* = 0.940, 95% CI 0.580–1.657) (Table 10). 

### 3.7. Access to Care

The mean number of in-person home visits during the initial home care episode (including recertification) for telehomecare patients was 5.0 nursing visits (SD 1.8) and 4.2 (SD 1.1) for usual care patients, *P* = .013. Over the entire six month study period, patients in the telehomecare group received on average 11 home visits (SD 8.9) and control group patients received on average 8 home visits (SD 4.6). This was significantly more in person contact for the telehomecare patients than the usual care patients (*P* < .001). Telehomecare patients were recertified for an additional episode of home care significantly more often than control group patients (24% compared to 9%, *P* = 0.003). The length of the initial home care episode for telehomecare patients was significantly longer at 54 days (SD 41) compared to usual care patients at 35 days (SD 23), *P* < 0.001. On average telehomecare patients who accepted the equipment received three video visits and used the monitoring devices 81% of the available days (SD-24, range 6%–100%). 

### 3.8. Patient Satisfaction

Patients in both groups were equally satisfied that they received enough home visits (89% of usual care group and 84% of telehomecare patients rated this item agree or strongly agree). But when asked specifically if they felt they were discharged too soon, 75% of usual care patients said agree or strongly agree while only 25% of telehomecare patients reported this (*P* = 0.03). In addition, 7% of usual care patients were not satisfied that they knew how to contact their nurse while no telehomecare patients reported this (*P* = 0.02). 

## 4. Discussion

By 30 days, telehomecare patients were readmitted 3% less often than usual care patients for all causes and 1% less for heart failure-related cause. By 60 days the overall difference in readmissions was 6% less for telehomecare patients. Although not statistically significant, Lubell states that decreasing readmissions by just 5% could save Medicare five billion dollars annually [44]. 

Differences in success rates with telehomecare may be due to patient and study site characteristics [16]. Two recent systematic reviews report that telehealth monitoring decreased the rate of heart failure readmissions by 21% [45], or 27% to 46% [19]. These reviews included all adult patients (18 and older) and either excluded home care patients [45] or combined studies with homebound and ambulatory patients [19]. Homebound status is a requirement for Medicare payment in the United States; so our study focused on older adult, homebound home care recipients, with a majority of African American race (65%), which may create a higher risk cohort than other studies. Soran and colleagues [46, 47] found no significant difference in readmission outcomes in a similar female, nonwhite, and older cohort of 315 subjects. A meta-analysis by Dellifraine and Dansky [20] reported statistically lower effect sizes in studies with mostly women *d* = 0.32 compared to *d* = 0.77 in studies with mostly men, *d* = 0.41 with the elderly compared to *d* = 0.61 with all adult subjects, and differences of *d* = 0.36 for mostly black compared to *d* = 0.65 for mostly white subjects. 

Soran and colleagues [47] suggest that differences in telehomecare study outcomes may arise from sites where heart failure treatments are already optimized. The control group 30-day HF readmission rate in our study was only 9% while the national 30-day HF readmission rate is nearly 24% [3]. Systematic reviews are showing decreases in HF readmission rates of 21–46% with telehealth [2, 8, 19, 45]. The low rate experienced at our study agency may have made it harder to show a difference. Additionally, the study was powered at a time when the readmission rate was 37%. 

The success of telehomecare interventions also depends on the context and skill with which they are applied. This study suffered from several problems that most likely impacted the ability to show a difference. The rate of patient acceptance of the technology was less than optimal. Nearly one-fourth of the sample refused to use or allow installation of the telehomecare equipment once it arrived in the home. Patients reported “feeling too sick to bother” with the technology. Strategies may include having the physician encourage the patient to do telemonitoring or involving family members to assist. The study targeted Medicare recipients making it an older sample, while findings indicated that the younger patients were more accepting of telehealth. Other studies experienced similar difficulties. Chaudhry et al. [17] had 14% of patients that did not use the system at all and Capomolla et al. [16] reported that 18% failed to use their system. Providing equipment that is simple, easy to set-up, and easy to use might help. Helping patients to see the benefit for them may increase motivation. Offering telehealth outside of the context of a study may also help as some patients did not want to be bothered with the obligation of follow-up interviews. Finally, buy-in from home care nurses to support and encourage the use of technology is a key facilitator. These issues decreased the size of the sample with exposure to the intervention resulting in a lack of power to show statistical significance. Other investigators have experienced these same barriers and more [11, 48–50]. 

Clinicians at the study site were not experienced with using telehomecare, but Chaudhry et al. [17] found no difference in outcomes in sites experienced with telehealth or not. Perhaps more important is the ability to respond quickly to changes in patient symptoms. Desai and Stevenson [51] recommend using an independent mid-level professional such as a nurse practitioner to provide timely treatment. In our study, communication flowed from the telehomecare nurse to the home care nurse, then to the primary physician using the telephone, which may have resulted in less than optimum responsiveness. Having a clinician who can provide changes in the medical treatment directly is likely to improve response time. 

Several home care policies and procedures created barriers to study goals. Decreasing in-person visits was difficult because the agency nurses were evaluated based on productivity defined as the number of completed in-person visits per day. To decrease visits would decrease their productivity as measured. Buy-in must exist from the top and middle managers, and within the nurses to ensure recognition for the time and effort spent on telehealth activities. Nurses felt pressured by middle managers to complete in-person visits to maintain productivity standards leaving less time to devote to telehealth activities. The agency did not recognize a video visit as part of their productivity; so there was no incentive for a nurse to eliminate an in-person visit. Further, if their “productivity” fell, they would be given a new admission therefore increasing the number of patients for whom they were responsible without added compensation. Agency management should recognize the workload contribution of telehomecare nurses and count the effort afforded to install, teach, monitor, and manage the clinical responses to the data. However, at this point the reimbursement incentives within homecare do not support this. 

Despite the study goal, and continuous reminders to the clinicians to be efficient and reduce the number of in-person visits using the technology, telehomecare patients received more in-person visits and were recertified for more home care than usual care patients. In most cases, intervention patients had more interaction with home health agency staff. Not only did telehealth patients have a field nurse, but also they had a telehealth nurse as well. Perhaps between the two nurses one or the other influenced recertification. Also, telehealth captures alterations in health status that may have gone unnoticed in the control group leading nurses to conclude that their patient was not stable enough for discharge. It is also possible that the telehealth nurse influenced the field nurse to recertify intervention patients as patients who utilized the technology liked using it and may have expressed this to the field or telehealth nurse, making recertification more common among this group. 

Most studies use telehealth technology in addition to home visiting [12, 15, 20, 52–55]. To our knowledge, only one published study [48] (since our study was designed) attempted to decrease home care visits using telehomecare and also was not successful. This nonrandomized study gave telehealth to patients who would accept it compared to those who refused. Only 3% used the system more often than weekly. When analyzing 15 high users, they found that home visits did decrease for 9 of the 15, but not for the majority of the samples. One possible explanation for more visits is the monitoring alerts clinicians to symptoms which may prompt them to assess the patient in person. Further study is needed to fully understand why telehomecare patients received more visits and more recertification. Financial incentives to nurses for using the technology more efficiently are also needed. 

Telehomecare patients expressed more satisfaction with home care than usual care patients in areas associated with access to care. They felt better prepared for how to contact their nurse and fewer expressed feeling discharged too soon. Given the fact that telehomecare patients were kept in home care longer, communicated both in-person and via video, and received more visits may explain the finding. In general telehealth studies report patient satisfaction with the technology [27, 56]. 

### 4.1. Limitations

This study was conducted in one home care agency in Southeastern Pennsylvania. The agency had no experience with telehomecare and therefore had to create new workflow procedures and generate enthusiasm for the new clinical program. Patients were limited to those 55 and older with a heart failure hospitalization within two weeks of enrollment and cognitively intact. Twenty-four percent of telehomecare group patients refused to use the equipment. The study sample was largely older, African-American females.

## 5. Future Research

Further research is needed to determine the ideal population for telehomecare effectiveness. Workflow must be carefully designed to support fidelity to the intervention and motivation of patients and nurses to use the equipment as prescribed. Protocols that support treatment parameters for rapid response need developed and tested. Financial incentives to use the technology efficiently are sorely needed. In addition, little is known about the ideal duration of monitoring or combination of case management, telephone use, and remote monitoring. 

## Figures and Tables

**Table 1:** Visit protocol for telehealth patients.

Week 1	2 in-home visits
Week 2	1 in-home; 1 video visit (nurses/patients collaborate on the plan of care)
Week 3	1 in-home; 1 video visit (nurses/patients collaborate on the plan of care)
Week 4	2 video visits
Week 5	2 video visits

Decision point	

Week 6	1 video visit; Nurses determine if it is time to discharge the patient or whether the patient could benefit from two more weeks of telehealth monitoring and teaching. If discharged, the nurse goes to the home (visit 5) to make the discharge visit, completes the discharge OASIS, and packs the telehealth equipment for return.
	If not discharged at week 6, the video nurse continues one video visit per week for weeks 7 and 8 and then goes into the home for the final visit to either close the case or recertify.

**Table 2:** Sociodemographic and baseline clinical characteristics of ITT subjects by treatment group.

Variable	Telemed (*N* = 101)			Control (*N* = 116)			*P*-value^1^
	*n*	%	****	*n*	%		

Gender							
Male	36	35.6		39	33.6		0.755
Female	65	64.4		77	66.4		
Ethnic Group							
Hispanic or Latino	0	0		2	1.7		0.185
Not Hispanic or Latino	101	100		114	98.3		
Race							
white	33	32.7		39	33.6		0.727
black or AA	66	65.3		75	64.7		
Asian	0	0		1	0.9		
Native Hawaiian or other pac. islander	0	0		0	0		
Am. Indian/Alaska native	1	1		1	0.9		
no response	1	1		0	0		
Marital Status							
Married	28	27.7		28	24.3		0.315
Widowed	35	34.7		51	44.3		
Separated	7	6.9		3	2.6		
Divorced	17	16.8		14	12.2		
Single	14	13.9		19	16.5		
Education							
Grades 1–4	1	1		0	0		0.770
Grades 5–8	9	8.9		11	9.6		
High school incomplete	21	20.8		27	23.5		
High school complete	49	48.5		43	37.4		
Post HS/Bus or trde school	3	3		4	3.5		
1–3 years college	8	7.9		15	13		
College completed	5	5		7	6.1		
Post graduate college	4	4		7	6.1		
No response	1	1		1	0.9		
Work history							
Full-time	0	0		0	0		0.282
Part-time	3	3		1	0.9		
Retired, not working	68	69.3		72	62.6		
Retired, BUT working	5	5		4	3.5		
Retired on disability	20	19.8		22	19.1		
Unemployed	4	4		12	10.4		
Never employed	0	0		3	2.6		
No response	1	1		1	0.9		
Income							
Less than $5,000	6	6		9	7.8		0.969
$5,000–$9,999	18	18		15	13		
$10,000–19,999	16	16		19	16.5		
$20,000–$39,999	14	14		15	13		
$40,000-or More	9	9		11	9.6		
Don't know	20	20		26	22.6		
No response	17	17		20	17.4		
Self-rate overall health at baseline							
Excellent	2	2		2	1.7		0.085
Very good	3	3		16	13.9		
Good	22	21.8		22	19.1		
Fair	47	46.5		50	43.5		
Poor	27	26.7		25	21.7		
Who lives with patient							
Spouse	23	22.8		26	22.6		0.905
Other relative	39	38.6		47	40.9		
Friend	4	4		2	1.7		
Other	1	1		1	0.9		
No one	34	33.7		39	33.9		
Avail. Of Primary Caregiver							
No	6	5.9		8	7		0.096
Yes	87	86.1		103	89.6		
Don't know	8	7.9		2	1.7		
No response	0	0		2	1.7		
# of overnight hosp. in past 12 months							
Not at all	8	7.9		7	6.1		0.935
One time	24	23.8		29	25.2		
Two or three	36	35.6		39	33.9		
More than three times	33	32.7		40	34.8		
# of physician or clinic visits past 12 months							
Not at all	2	2		4	3.5		0.331
once	3	3		0	0		
2-3 times	8	7.8		9	7.8		
4–6 times	31	30.7		30	26.1		
more than 6 times	57	56.4		72	62.6		
Severity of illness from home care OASIS							
Asymptomatic, no treatment needed at this time	0	0		0	0		0.114

Symptoms well controlled with current therapy	0	0		2	1.8		
Symptoms controlled with difficulty, affecting daily functioning; patient needs ongoing monitoring	52	51.5		72	63.2		
Symptoms poorly controlled; patient needs frequent adjustment in treatment and dose monitoring	40	39.6		30	26.3		
Symptoms poorly controlled; history of re-hospitalizations	9	8.9		10	8.8	
Variable	*N*	mean	SD	*N*	mean	SD	*P*-value^2^

Age (yrs.)	101	71.3	10.2	116	73.5	9.6	0.092
CHF (months)	101	60.7	67.7	115	61.5	71.6	0.935
# Concomitant Medications	95	11.3	4.6	113	10.0	3.4	**0.020**
# Comorbid Conditions	101	6.8	4.0	116	6.0	4.0	0.145

Notes: ^1^Chi-square test ^2^ *T*-Test, one patient in the control group had an incomplete baseline interview and medication counts were unobtainable for nine patients enrolled from hospitals outside the health system.

**Table 3:** Results from cox proportional hazards modeling of time to first all-cause readmission or death.

Variable		Estimate	SE	HR	95% CI		*P*
group	TH	−0.204	0.205	0.816	0.546	1.22	0.319
age		−0.007	0.010	0.993	0.974	1.01	0.508
nmed		0.040	0.025	1.041	0.992	1.09	0.106

SE: Standard Error; HR: Hazard Ratio; TH: Telehealth.

**Table 4:** Number and percentage of readmissions by time period and group.

All-cause readmission				
Group	0–30 days	31–60 days	61–120 days	121–180 days
	*n* (%)	*n* (%)	*n* (%)	*n* (%)

Control	22 (19%)	22 (19%)	27 (23%)	26 (22%)
Telehealth	16 (16%)	20 (20%)	20 (20%)	23 (23%)

Heart failure-related readmission				

Group	0–30 days	31–60 days	61–120 days	121–180 days
	*n* (%)	*n* (%)	*n* (%)	*n* (%)
Control	10 (9%)	4 (3%)	13 (11%)	9 (8%)
Telehealth	8 (8%)	9 (9%)	9 (9%)	8 (8%)

**Table 5:** Results from GEE model for readmissions.

All cause							Heart failure related				
		Exp (B)	SE	95% CI		*P*	Exp (B)	SE	95% CI		*P*

Period	31–60	1.070	0.205	0.716	1.600	0.743	0.737	0.342	0.377	1.442	0.373
Period	61–120	0.686	0.189	0.474	0.993	0.046	0.605	0.302	0.335	1.093	0.096
Period	121–180	0.791	0.209	0.525	1.191	0.261	0.526	0.343	0.269	1.031	0.061
group	TH	0.790	0.197	0.537	1.162	0.230	0.992	0.295	0.556	1.769	0.978
age		0.989	0.010	0.970	1.008	0.245	0.986	0.017	0.953	1.021	0.427
nmed		1.018	0.023	0.972	1.065	0.450	1.004	0.039	0.931	1.083	0.917

SE: Standard Error, TH: Telehealth Group; Baseline categories: 0–30 days for Period and Control for Group.

**Table 6:** Mean and SD of hospital days by time period and group.

All-cause readmission				
Group	0–30 days	31–60 days	61–120 days	121–180 days
	Mean (SD)	Mean (SD)	Mean (SD)	Mean (SD)

Control	1.41 (4.05)	1.07 (3.07)	2.41 (7.72)	2.02 (5.22)
Telehealth	0.91 (2.49)	1.17 (3.21)	1.49 (4.53)	2.03 (5.28)

HF related-readmission				

Group	0–30 days	31–60 days	61–120 days	121–180 days
	Mean (SD)	Mean (SD)	Mean (SD)	Mean (SD)

Control	0.38 (1.38)	0.11 (0.72)	0.88 (3.13)	0.32 (1.37)
Telehealth	0.48 (1.75)	0.41 (1.55)	0.68 (3.48)	0.49 (1.94)

**Table 7:** Results from GEE model for hospital days.

All-cause							Heart failure-related				
		Exp (B)	SE	95% CI		*P*	Exp (B)	SE	95% CI		*P*

Period	31–60	0.911	0.270	0.536	1.548	0.731	0.587	0.361	0.289	1.191	0.140
Period	61–120	0.831	0.254	0.505	1.366	0.464	0.837	0.381	0.396	1.768	0.641
Period	121–180	0.871	0.264	0.519	1.461	0.601	0.467	0.370	0.226	0.966	0.040
group	TH	0.758	0.246	0.468	1.227	0.260	1.245	0.344	0.634	2.445	0.524
age		0.973	0.013	0.949	0.998	0.035	0.979	0.019	0.944	1.016	0.262
nmed		1.003	0.029	0.947	1.061	0.931	1.002	0.041	0.924	1.086	0.968

SE: Standard Error, TH: Telehealth Group; Baseline categories: 0–30 days for Period and Control for Group.

**Table 8:** Results from cox proportional hazards modeling of time to ED use.

Variable		Estimate	SE	HR	95% CI		*P*
group	TH	−0.248	0.208	0.78	0.519	1.171	0.231
age		−0.008	0.01	0.992	0.973	1.012	0.448
nmed		0.029	0.025	1.029	1.029	0.98	0.245

SE: Standard Error; HR: Hazard Ratio; TH: Telehealth.

**Table 9:** Number and percent of ED visits by time period and group.

Group	0–30 days	31–60 days	61–120 days	121–180 days
	*n* (%)	*n* (%)	*n* (%)	*n* (%)

Control	12 (10%)	4 (3%)	14 (12%)	13 (1%)
Telehealth	10 (10%)	7 (7%)	14 (14%)	13 (13%)

**Table 10:** Results from GEE model for ED visits.

Parameter		Exp (B)	SE	95% CI		*P*
Period	31–60	0.480	0.361	0.237	0.973	0.042
Period	61–120	0.660	0.253	0.402	1.083	0.100
Period	121–180	0.640	0.289	0.364	1.127	0.122
group	TH	0.980	0.268	0.580	1.657	0.940
age		0.965	0.014	0.938	0.992	0.013
nmed		1.015	0.033	0.951	1.083	0.661

E: Standard Error, TH: Telehealth Group; Baseline categories: 0–30 days for Period and Control for Group.
